# Molecular Insights into Radiation Effects and Protective Mechanisms: A Focus on Cellular Damage and Radioprotectors

**DOI:** 10.3390/cimb46110755

**Published:** 2024-11-09

**Authors:** Blanca Ibáñez, Ana Melero, Alegría Montoro, Nadia San Onofre, Jose M. Soriano

**Affiliations:** 1 Food & Health Laboratory, Institute of Materials Science, University of Valencia, 46980 Paterna, Spain; blancaibanezdepedro@gmail.com (B.I.); sanonofre.nadia@gmail.com (N.S.O.); 2 Department of Pharmacy and Pharmaceutical Technology and Parasitology, Faculty of Pharmacy, University of Valencia, 46100 Burjassot, Spain; ana.melero@uv.es; 3 Service of Radiological Protection, Clinical Area of Medical Image, University and Polytechnic La Fe Hospital, 46026 Valencia, Spain; montoro_ale@gva.es; 4 Biomedical Imaging Research Group GIBI230, Health Research Institute (IISLaFe), University and Polytechnic La Fe Hospital, 46026 Valencia, Spain; 5 Department of Community Nursing, Preventive Medicine and Public Health and History of Science, University of Alicante, 03690 Alicante, Spain; 6 FoodLab Research Group, Faculty of Health Sciences, Universitat Oberta de Catalunya, Rambla del Poblenou 156, 08018 Barcelona, Spain; 7 Joint Research Unit on Endocrinology, Nutrition and Clinical Dietetics, Health Research Institute La Fe, University of Valencia, 46026 Valencia, Spain

**Keywords:** ionizing radiation, DNA damage, radioprotectors, reactive oxygen species (ROS), radiation-induced damage, radiotherapy, antioxidants, free radicals, radiation protection, molecular mechanisms

## Abstract

Ionizing radiation has been a critical tool in various fields, such as medicine, agriculture, and energy production, since its discovery in 1895. While its applications—particularly in cancer treatment and diagnostics—offer significant benefits, ionizing radiation also poses risks due to its potential to cause molecular and cellular damage. This damage can occur through the direct ionization of biological macromolecules, such as deoxyribonucleic acid (DNA), or indirectly through the radiolysis of water, which generates reactive oxygen species (ROS) that further damage cellular components. Radioprotectors, compounds that protect against radiation-induced damage, have been extensively researched since World War II. These agents work by enhancing DNA repair, scavenging free radicals, and boosting antioxidant defenses, thereby protecting healthy tissues. Furthermore, some radioprotective agents also stimulate DNA repair mechanisms even after radiation exposure, aiding in recovery from radiation-induced damage. This article explores the molecular mechanisms of radiation-induced damage, focusing on both direct and indirect effects on DNA, and discusses the role of radioprotectors, their mechanisms of action, and recent advancements in the field. The findings underscore the importance of developing effective radioprotective strategies, particularly in medical and industrial settings, where radiation exposure is prevalent.

## 1. Introduction

Ionizing radiation has been widely used since 1895, following the discovery of X-rays by Wilhelm Roentgen [1]. Over the past century, its applications have greatly expanded, particularly in fields such as medicine, agriculture, energy production, and food preservation. In medicine, ionizing radiation is critical for both diagnostic imaging, such as X-rays and CT scans, and therapeutic treatments, particularly in cancer radiotherapy [2]. In agriculture, radiation is used to sterilize pests and extend the shelf life of food products, contributing to food safety [3]. Similarly, in energy production, radiation plays a vital role in nuclear power generation [4]. However, despite its extensive benefits, the widespread use of ionizing radiation poses significant risks to living organisms due to its potential to cause harmful molecular and cellular damage [5]. Ionizing radiation interacts with biological tissues through two primary mechanisms: direct and indirect [6]. Direct effects occur when radiation directly ionizes biological molecules, such as DNA, causing breaks in single or double strands. While DNA is a primary target, other biomolecules, including proteins and lipids, can also be directly affected, leading to a range of molecular and cellular damage. These breaks can result in mutations, genomic instability, or cell death if not properly repaired [7]. Indirect effects, on the other hand, arise when radiation interacts with water molecules within cells, producing reactive oxygen species (ROS) and reactive nitrogen species (RNS). These ROS can damage cellular components, including lipids, proteins, and nucleic acids, further exacerbating the harmful effects of radiation exposure [8]. The combination of these mechanisms can result in a wide range of biological outcomes, from acute tissue damage to long-term effects such as carcinogenesis. This is particularly concerning in medical and industrial settings, where workers and patients may be exposed to ionizing radiation. Given these risks, it is critical to develop protective strategies to mitigate radiation-induced damage. Over the years, scientists have explored the use of radioprotectors, which are agents designed to protect normal tissues from the harmful effects of radiation [9]. This manuscript aims to provide a detailed exploration of the molecular mechanisms of radiation-induced damage, with a specific focus on the direct and indirect effects of radiation on cellular components, and to assess the biological effects of ionizing radiation by examining how it impacts cellular processes at the molecular level. Furthermore, the role and mechanisms of radioprotectors are evaluated, discussing how these agents function to mitigate or prevent radiation-induced damage and highlighting recent advancements in radioprotection strategies. To conduct this review, a literature search [10] was carried out across two major databases: PubMed and Web of Science. The search strategy employed a combination of Medical Subject Headings (MeSHs) and keywords, such as “radiation effects” OR “DNA damage” OR “radioprotectors”; AND “ionizing radiation”, to identify relevant studies focused on radiation-induced molecular damage and protective mechanisms. The inclusion criteria were broad, emphasizing articles that provided significant insights into the biological effects of ionizing radiation, particularly those related to DNA damage and radioprotective agents. The articles reviewed included primary research studies, such as randomized controlled trials, cohort studies, and retrospective analyses, as well as relevant reviews and meta-analyses that offered a comprehensive understanding of the topic. Studies that did not meet the inclusion criteria, such as abstracts, ongoing studies, articles without full-text availability, and non-English publications, were excluded. Two teams of paired reviewers (B.I./J.M.S; A.M./N.S.O.), with expertise in medical and health evaluations and training in research methodology, independently screened the titles, abstracts, and full texts for eligibility. Any disagreements were resolved by a third researcher (A.Mo).

## 2. Molecular Mechanisms of Radiation-Induced Damage

### 2.1. Direct and Indirect Effects on DNA

**Figure 1:**
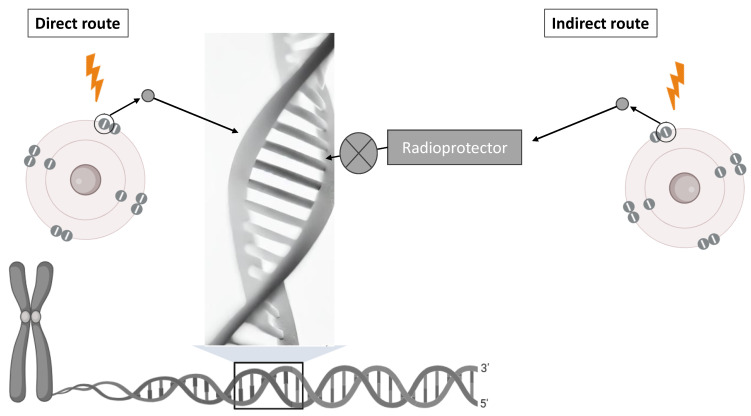
Direct (radical-induced primary damage) and indirect (reactive free radicals, that is, ^•^OH, produced following radiolysis of water) route action of ionizing radiation. Radiation protection of radioprotector is carried out in the indirect route. The X symbol indicates the pathway inhibited by this radioprotector. Figure created using Render (Render Inc., San Francisco, CA, USA).

The direct effect occurs when the interaction of radiation occurs with a biological macromolecule, altering atomic structures and causing chemical and physical changes. An example of a direct effect is a break in the DNA double helix caused by the ionization of the molecule itself after receiving ionizing radiation [11] (Figure 1). In fact, the break in the DNA strand is not caused by the initial incident radiation, such as X-rays, but by the electrons that this beam has released through ionization [12]. Interestingly, the mean free path for electrons with energies of a few tens of eV, defined as the average distance traveled between two successive interactions, is a few nanometers, coinciding with the width and dimensions of the DNA strand [13]. Consequently, its effect is much more significant than that of the initial radiation itself [14]. Although the most studied direct effect of radiation is the interaction with DNA, other biomolecules can also be damaged by the path of radiation [15]. The direct ionization process of biomolecules (RH) follows the following reactions [16,17,18]:

RH + Ionizing radiation → RH^+^ + e^−^

This is followed by dissociation:

RH^+^ → R^•^ + H^+^

In all cases, free radicals (R^•^) are produced and remain.

In radiation biology, it is essential to distinguish between high- and low-Linear Energy Transfer (LET) particles, as the nature of DNA damage varies significantly between the two. High-LET particles, such as carbon ions, produce dense ionization tracks, leading to complex, clustered DNA damage that is challenging for cellular repair mechanisms to resolve. In contrast, low-LET particles, like X-rays and gamma rays, produce more dispersed damage with single-strand breaks that cells can often repair. This difference underscores the distinct mechanisms of direct and indirect damage, with high-LET radiation causing more direct, localized damage in cellular structures [19].

On the other hand, the indirect effect (Figure 1) arises from a series of chemical reactions between cells and free radicals, or other products generated by the radiation itself [20]. Thus, the indirect effect is caused by an interaction of the radiation with the molecules of the organism, mainly water molecules [21]. Within this indirect effect, we would see the radiolysis of cellular water and the generation of ROS and RNS by the enzyme nitric oxide synthase (NOS), causing damage and breaks in DNA and RNA [22]. An example of an indirect effect would be a break in the DNA double helix due to the attack of OH radicals on the sugars of the DNA, occurring at a later time [23].

Due to the abundance of water in our bodies, the indirect effect predominates over the direct effect. It is estimated that DNA breakage occurs 40% due to direct interactions and 60% due to indirect processes [11]. As radiation passes through our bodies, both mechanisms are present, triggering a series of biochemical and molecular responses in the tissue. These responses may lead to cell repair, permanent physiological changes, or even cell death. Cell death can occur either due to the inability of the cell to continue dividing or the loss of its specific function [24].

The most significant indirect phenomena of ionizing radiation are the radiolysis of water and its resulting products [25]. These reactions occur in four temporal phases (physical, pre-chemical or physicochemical, chemical, and biological) within a specific timeline, as shown below [26].

In the physical phase, as a result of the passage of ionizing radiation through matter, ionized H_2_O^+^ and excited H_2_O* molecules are produced [27]. Secondary electrons are also generated in the matter [18]. However, most of these possess sub-excitatory energy, decelerate quickly, and do not cause alterations in atomic structures, though they do at the cellular level, as previously mentioned regarding direct DNA damage [14]. Depending on the amount of energy absorbed from the ionizing radiation, the ionization or excitation of molecules will occur [28]. In the ionization process, the transferred energy must be greater than the electron’s binding energy, which depends on the atomic or molecular orbital in which it is located [29]. This causes the electron to leave the atom, creating a positive ion [13].

H_2_O + Ionizing radiation → H_2_O^+^ + e^−^

In the excitation process, the energy transferred by the interaction of the incident radiation with an atomic electron is insufficient to cause ionization; the impacted electron does not leave the atom but may move to a higher-energy orbit [30]. Since physical systems tend to be in the lowest energy configuration, such an excited electron will return the energy in the form of electromagnetic radiation, in what are called radiative transitions, or it will degrade into heat in non-radiative transitions [12,31].

H_2_O + Radiation → H_2_O*

For pre-chemical or physicochemical phase, the three species initially produced by the radiation (ionized molecule, excited molecule, and free electrons) are extremely unstable and reorganize in various ways [16]; these species can return to their initial state either by the ionized molecule absorbing the free electron or by the excited molecule emitting radiation [21]. However, they can also lead to reactions that result in new molecular systems. For example, the positive ion can react with a new water molecule, resulting in the following:

H_2_O^+^ + H_2_O → H_3_O^+^ + OH^•^

On the other hand, the excited water molecule releases its energy by losing an electron, thus becoming an ion, or through molecular dissociation [32]. Both reactions are shown below [33]:

H_2_O^+^ + e^−^ ← H_2_O^•^ →H^•^ + OH^•^

Finally, secondary electrons migrate, losing energy through the excitation of water molecules [26]. As they lose this energy, they become thermal electrons (electrons that lose energy until they have kinetic energy comparable to that of gas molecules due to thermal agitation) [34]. In this state, the electron becomes surrounded by a layer of water molecules, forming a true hydrated electron in aqueous solution [30].

For the chemical phase, during this third stage, the four chemically reactive species (aqueous electron; H^•^ and OH^•^, which are free radicals; and H_2_O^+^) diffuse and react with each other or with the environment until all reactions are completed [25]. The main reactions that occur in water during this stage are as follows:

OH^•^ + ^•^OH → H_2_O_2_

Hydrated electron + OH → OH^−^

H^•^ + ^•^OH → H_2_O

H_3_O^+^ + hydrated electron → H^•^ + H_2_O

2 hydrated electrons + 2 H_2_O → H_2_ + 2 OH^−^

Hydrated electron + H^•^ + H_2_O → H_2_ + OH^−^

H^•^ + H^•^ → H_2_

As observed in the three previous stages, the result of the interaction with water is the production of a series of reactive species, among which we find three free radicals that continue to react, producing different ions and molecular products [35]. Many studies show that, quantitatively, the most important species produced from the radiolysis of water are aqueous electrons (e^−^_aq_), H^•^ (hydrogen radical), OH^•^ (hydroxyl), H_2_, and H_2_O_2_ (hydrogen peroxide) [36]. The H^+^ and OH− ions can combine to produce a new water molecule or chemically react, affecting the surrounding molecules [37]. The probability of them recombining is higher than that of them reacting and causing cellular damage. Nevertheless, their high reactivity and mobility in the medium allow their action to propagate through it, potentially causing damage in locations far from their origin [38]. Furthermore, as can be observed, the combination of free radicals with each other produces different molecular compounds, which can sometimes be toxic to the cell, such as hydrogen peroxide, which will cause further damage [39].

Another important reaction that occurs in the presence of oxygen [40] is the conversion of H atoms and aqueous electrons into peroxide or hydroperoxyl radicals, reactive oxygen species that can cause direct biological damage or combine with another hydroperoxyl radical to form hydrogen peroxide and oxygen [41,42].

H^•^ + O_2_ → HO^•^_2_

HO^•^_2_ + HO^•^_2_ → H_2_O_2_ + O_2_

In biological systems, organic radicals (R^•^) are also formed by hydrogen atom abstraction that is initiated, for example, by OH radicals [43]. These organic radicals react quickly with oxygen to produce peroxides (RO_2_^•^), which are more oxidizing than the molecules from which they originate [44]. These peroxides can take a hydrogen atom from other molecules and form hydroperoxides (ROOH), a reaction involved in lipid peroxidation [45].

Lastly, ionizing radiation can also stimulate the inducible NOS enzyme, leading to the production of large amounts of nitric oxide (NO). Although NO is chemically inert, it has the ability to react with O_2_, producing a peroxynitrite anion (ONOO¯), which is highly reactive and capable of attacking various cellular targets, such as lipids, proteins, DNA bases, and thiols [46].

The production of these ROS and RNS is highly important because recent research shows that although the direct effects of ionizing radiation can be observed in cells shortly after exposure, the oxidative changes caused by these reactive species can continue for days and months after exposure, as they are generated continuously [47]. Additionally, other studies support the idea that these oxidative processes not only occur in directly irradiated cells but also in subsequent generations [48]. Moreover, the effects of oxidative stress can be observed not only in directly irradiated target cells but also in neighboring cells that were not exposed to radiation, through mechanisms such as non-targeted effects (NTE), including bystander effects. These occur via intercellular communication and the secretion of specific molecules by irradiated cells, which interact with non-exposed cells [49].

In the biological phase, biological effects and cellular damage occur. As previously explained, it is generally assumed that the biological effects on cells result from the direct or indirect action of radiation [50]. Direct action occurs through the initial impact of ionizing radiation on biological structures, where they absorb the energy, producing ionizations and excitations in the atoms of the molecules where the interaction occurred (similar to water radiolysis) [51]. Indirect effects are mainly caused by free radicals and other radiation products generated in the previously explained process [52]. The biological damage can be somatic or hereditary, it can be deterministic (non-stochastic) or stochastic (random), or it can occur in target molecules or in cells and components that do not function as targets [53]. After this biological damage occurs, a series of biochemical and molecular signaling mechanisms follow, culminating in the repair of the damage or possibly resulting in permanent physiological changes and even cell death [54]. Among the effects on molecules and biological structures, the following stand out:

- Effects of radiation on proteins. Cellular proteins are polypeptides with very important biological functions. Among other roles, they form part of structural elements or have enzymatic activity necessary for many processes, such as metabolism. They are stabilized by various forces, such as hydrogen bonds, and are organized into different structural levels. The structure and organization are essential for their function. If a cell is irradiated, these proteins can undergo changes due to the direct action of radiation, where the protein itself can become ionized, typically occurring in the carbon of glycine or in the sulfur of cystine or cysteine residues. They can also undergo changes through indirect action, such as the attack of OH radicals on aromatic and sulfur-containing amino acids, like methionine, cysteine, or cystine [55]. Additionally, other amino acids may be attacked by hydrated electrons produced indirectly. Following these modifications, protein denaturation is the main consequence [56]. This process involves a reversible or irreversible alteration of the protein’s most stable structure. By modifying its structure, its functionality and biological activity are also altered, potentially causing changes in transport, catalysis, metabolism regulation, and structural support of the cell. If the denaturation process is not too severe, renaturation may occur after the stress caused by ionizing radiation disappears, restoring the original conformation [57].
- Effects of radiation on nucleic acids. Nucleic acids are key elements in the biological effects of ionizing radiation due to their functions as carriers of genetic information (DNA), protein production (ribonucleic acid, RNA), and controllers of gene expression (microRNA, miRNA) [58]. Again, damage to nucleic acids can be direct or indirect through free radicals. It should be noted that the alteration or destruction of even a single base in the nucleic acid can have significant biological importance if that base was located at a critical point encoding a fundamental process [59]. Additionally, different bases have varying sensitivity to radiation, with pyrimidine bases being 100 times more sensitive to ionizing radiation than purine bases. Besides base damage, other alterations can occur, such as single- or double-strand breaks in the nucleic acid. DNA lesions can be classified into five groups: single-strand break (SSB), double-strand break (DSB), base damage, DNA–protein crosslinks, or DNA-DNA crosslinks. It is estimated that 1 Gy of low-Linear Energy Transfer (LET) ionizing radiation leads to 1000 single-strand breaks, 40 double-strand breaks, 500 base damages, and 150 DNA–protein crosslinks [60].
- Effects of radiation on biological membranes. Biological membranes control the transport of materials between the inside and outside of a cell, or between different cells, and play an important structural role, particularly in bioenergetic processes. Different biological membranes exhibit varying degrees of radiosensitivity, meaning that the effects of ionizing radiation on membranes can be detected at different radiation doses [61]. For example, protein synthesis in the endoplasmic reticulum or photosynthesis in chloroplasts is only slightly affected by radiation, as these systems are generally more resistant to ionization. In contrast, the mitochondrial membrane, where oxidative phosphorylation occurs, is considerably more sensitive to radiation. Many of the effects of radiation on biological membranes are due to the phenomenon of lipid peroxidation. Free radicals generated by the indirect action of ionizing radiation, including ROS, organic radicals, or RNS, cause lipid peroxidation, leading to the oxidation of polyunsaturated lipids and damaging membrane proteins. The formation of peroxides in the membrane results in structural changes that disrupt biochemical functions, alter permeability (causing hyperpolarization through improper activation of sodium–potassium channels), and interfere with active transport processes. One of the leading hypotheses suggests that these structural changes can cause irreversible damage and serve as initial triggers for apoptosis in irradiated cells [62].

### 2.2. Biological Effects of Ionizing Radiation

Primarily, these are divided into two groups:

- Somatic and hereditary effects. The effects that radiation has on living organisms can be classified according to different criteria. When the effects only impact the health of the irradiated individual, they are called somatic effects [63]. Among these, leukemia and other types of cancer (for example, lung cancer or thyroid cancer), shortened lifespan, or the development of cataracts are notable [64]. In contrast, when the effects also affect the offspring of the irradiated person, they are called hereditary effects [65]. Genetic or hereditary effects occur because ionizing radiation is capable of altering the genetic information contained in germ cells or the zygote, which are the only cells capable of transmitting alterations to future generations [66].
- Deterministic and stochastic effects. In addition to the distinction between somatic and hereditary effects, the effects can also be differentiated between deterministic (non-stochastic) and stochastic or probabilistic effects. Deterministic or non-stochastic effects are those caused directly by the absorbed dose, with no other factor being the cause of the effect, so the severity of the effect depends on the amount of dose absorbed [67]. These effects are due to the death of a relatively large number of cells in a tissue or organ [68]. They are the inevitable consequence of exposure to high levels of ionizing radiation. Examples include the production of erythema after irradiation, anemia, or the late onset of cataracts and acute radiation syndrome (ARS) [69]. For deterministic effects to occur, the dose must reach a certain threshold, below which these effects do not manifest [70]. Furthermore, these deterministic effects are divided into two groups:
  o At the cellular level. Considering that the mechanism through which deterministic effects occur is cell death, it is important to clarify what is meant by cell death, as this term has different meanings for different types of cells [71]. For differentiated cells, which do not proliferate, death means the loss of the function for which they were specialized, such as the loss of the ability to transport oxygen in the case of red blood cells [72]. However, for dividing cells, cell death implies that they have lost the ability to carry out division. Thus, after radiation exposure, an undifferentiated cell may be physically present and appear intact but has lost its ability to undergo successive divisions [73].
  o At the tissue level. Table 1 provides a summary of the main deterministic effects produced in different organs and tissues of the body after acute exposure to both low- and high-LET radiation. It also indicates the main causes of the effects, threshold doses, and doses that lead to severe deterministic effects [74].

On the other hand, the stochastic effects of ionizing radiation are those that may occur randomly due to radiation exposure when molecular damage has been poorly repaired, resulting in modifications to the affected cells [75]. This means that after irradiation (typically at low doses), reactions that may be related to radiation exposure can emerge over time [76]. For instance, exposure to ionizing radiation increases the probability of developing leukemia or malformed fetus (somatic effects) or the possibility of transmitting mutations to offspring (hereditary effect) [77]. However, it is impossible to definitively confirm that a specific case of leukemia or a malformed fetus is due to the received irradiation [78]. As the equivalent dose increases, so does the probability of one of these stochastic effects occurring. It is believed that there is no threshold dose for stochastic effects, and the probability of occurrence is proportional to the dose received [79]. In contrast, deterministic effects do have a threshold dose, below which these effects do not manifest [80]. Today, it is understood that the most relevant somatic stochastic effect after exposure to low radiation doses is the development of cancer [81]. The transition from a normal cell to a malignant cell is a complex process that involves various changes, the exact nature of which depends on the type of cell, the mechanism of action of the carcinogen involved, and the type of cancer that develops [82]. In medicine, the word “cancer” is used generically to refer to a group of diseases with more than a hundred distinguishable clinical forms, with different biological behaviors and clinical manifestations, encompassing over a thousand histopathological varieties [83]. Despite this diversity, various general models have been developed to describe the carcinogenic process, with the multistage model being the most accepted [84]. This model predicts that cancer arises as a consequence of a series of events that may be entirely independent, but are often linked, and may even be mediated by the same agent. The multistage model posits that cancer development occurs in four stages: initiation, conversion, promotion, and progression [85].

## 3. Role and Mechanisms of Radioprotectors

The concept of radioprotectors emerged after World War II, as part of efforts to shield humans from the potential consequences of nuclear warfare. Early research by Patt [86] demonstrated that certain compounds, such as cysteine, could protect experimental animals from the lethal effects of radiation. Since then, extensive research has focused on developing more effective and less toxic radioprotective agents [87]. These agents have become especially important in cancer treatment, where they are used to protect healthy tissues during radiotherapy, while allowing radiation to target tumor cells [88]. Radioprotectors work primarily by preventing radiation-induced damage through mechanisms such as scavenging free radicals, reducing oxidative stress, and boosting intrinsic antioxidant defenses. In contrast, mitigators, which are administered after radiation exposure, focus on enhancing DNA repair pathways and other cellular recovery processes to reduce the severity of radiation-induced damage [89]. While many compounds have been synthesized and tested for their radioprotective properties, developing an ideal radioprotector remains challenging [90]. Such an agent would need to provide comprehensive protection across various tissues, have minimal toxicity, and be easy to administer [91]. Additionally, it should not interfere with the therapeutic efficacy of radiation in cases like cancer treatment, where selective damage to tumor cells is the goal [92]. Ionizing radiation has been used since 1895, following Roentgen’s discovery, and its application has continuously expanded across society. Today, ionizing radiation is increasingly used in fields like agriculture, medicine, energy generation, and food preservation [93]. These advancements offer numerous benefits, but it is crucial to consider the potential harmful effects, such as the possible damage that ionizing radiation can cause to living organisms [94]. This risk has highlighted the need to protect populations from the unwanted effects of potential or accidental radiation exposures [95]. In the case of potential exposures, the rise in nuclear-energy use has increased the risk of nuclear accidents, as well as public concerns about terrorist attacks involving radioactive materials—often without justified cause [96]. In this context, developing agents to minimize or prevent adverse radiation effects in cases of war or radiological accidents is highly relevant [97]. Advances in science, particularly in understanding biological effects, have paved the way for developing agents that reduce radiation-induced damage, enhancing protection against the harmful effects of ionizing radiation, such as radioprotectors [98]. The first attempt to use chemical compounds as radioprotectors to mitigate radiation’s harmful effects occurred after World War II, motivated by the need to protect humans from nuclear weapons. Patt [86] was the first to investigate the effects of cysteine in rats exposed to lethal doses of X-rays. Over the last 60 years, driven by the clinical demand for effective radioprotectors, numerous compounds have been synthesized and studied to find the most effective ones with minimal toxicity [99]. Table 2 shows a list of desired characteristics for the ideal radioprotector.

In fact, radioprotectors are compounds that modify biological responses to radiation and include both chemical and natural compounds [87]. According to Urtasun [100], radioprotectors protect normal cells from radiation damage without protecting tumor cells. Vasin [101] classified anti-radiation drugs into three categories based on their mode of action: (i) drugs suppressing initial radiation symptoms, (ii) drugs detoxifying early-stage exposure, and (iii) drugs assisting in radionuclide absorption or elimination. A little later, Nair [102] classified radioprotective agents into three categories: (i) Adaptogens are non-toxic stimulants of radioresistance, acting as natural protectors that provide chemical protection against low levels of ionizing radiation. They are generally extracted from plants and other natural sources and have lower toxicity [95]. (ii) Absorbents are agents that protect against internal damage (internal dose) resulting from the ingestion of radionuclides. They prevent the uptake of radioactive iodine by the thyroid glands and the absorption of radionuclides like ^137^Cs, ^90^Sr, and ^239^Pu [103]. (iii) Radioprotectors include antioxidant compounds, such as certain myelo-, entero-, and cerebro-protectors, and others that contain sulfhydryl groups [104]. According to Stone [105], various classifications of radiation-protecting agents have been proposed in recent years, and many of them are now widely accepted by radiobiologists. He suggested that these agents can be categorized as radioprotectors, mitigators, and agents for treating the side effects of radiation [106]. The first term refers to prophylactic agents administered before irradiation [107]. The second group includes agents administered after exposure but before the damage manifests [108]. Finally, agents for treating radiation side effects are administered after the appearance of clinical symptoms [109].

Seed [110] defines a radioprotector as any agent or medicinal product applied before or during radiation exposure that actively prevents or limits damage at the molecular, cellular, tissue, organ, or systemic level. Many researchers define and classify radioprotective agents based on their mechanism of action, utility, route, and timing of administration [87]. Traditionally, they are classified into two main categories: (i) pre-irradiation agents, known as radioprotectors, which are administered before exposure to prevent radiation damage; and (ii) post-irradiation agents, or mitigators, which are administered after exposure to stimulate recovery from radiation-induced damage [80].

Radioprotective substances act through one or more molecular mechanisms that target various aspects of cellular damage. These mechanisms include (i) increasing intrinsic antioxidants, like glutathione; (ii) chelating metals to prevent oxidative stress; (iii) enhancing DNA repair and promoting cellular recovery; (iv) improving the anti-inflammatory response; (v) reducing the generation of reactive oxygen and nitrogen species; (vi) stimulating DNA-binding proteins and inducing chromatin compaction; (vii) reducing lipid peroxidation and protein oxidation; (viii) inducing hypoxia to limit free radical formation; (ix) inducing cell-cycle arrest to prevent propagation of damage; (x) enhancing cell proliferation for tissue repair; (xi) increasing free radical sequestration; (xii) stabilizing cytoplasmic and mitochondrial membrane potential; and (xiii) modulating the expression of proteins involved in apoptosis [111].

Among these mechanisms, several key actions should be highlighted:

- Suppression of reactive species formation: Some pharmacological agents act as radioprotectors by interfering with oxygen distribution in irradiated tissues, inducing local hypoxia in cells and tissues. Oxygen is required for the formation of many free radicals, such as ROS, so limiting its availability reduces the formation of harmful chemical species. For example, sulfhydryl compounds (RSH) undergo oxidation with molecular oxygen, chemically or biochemically consuming it. This mechanism provides radioprotection by preventing oxygen from generating new free radicals [112].
- Detoxification of radiation-induced species: These substances can significantly reduce the damage caused after exposure. Many can inactivate OH˙ and O˙ radicals, which are responsible for radiation-induced indirect damage. Sulfhydryl compounds, for example, react with reactive species due to their chemical affinity for OH˙ groups, as shown in the following reaction [113]:

RSH + OH˙ = RS + H_2_O

This reaction inactivates OH˙ radicals, preventing radiation-induced cell lethality. Another mechanism for eliminating aqueous free radicals is increasing antioxidant enzymes like glutathione reductase, glutathione peroxidase, superoxide dismutase, and catalase [103]. In addition to scavenging free radicals before they interact with critical cellular components, radioprotectors can also donate hydrogen atoms to stabilize these components, repairing biochemical lesions [114].

- Target stabilization: Radioprotectors can interact with various cellular targets, including DNA, proteins, and lipids. This interaction stabilizes these molecules and prevents radiation damage. For example, aminothiols like cysteamine bind to cellular structures, providing radioprotection [99]. Other molecules, such as spermidine, are also effective in stabilizing cell membranes and other critical components [115].
- Reinforcement of recovery and repair systems: Radiation can damage multiple cellular components, including DNA, proteins, and lipids. Endogenous radioprotective substances have been studied for their role in cellular recovery after radiation exposure. Many, such as thiols, are involved in the repair of damage across different biomolecules, including single-strand breaks in DNA and the restoration of oxidized proteins and lipids [116].

## 4. Conclusions

Ionizing radiation, while invaluable in fields such as medicine and energy, poses significant risks to living organisms by causing direct and indirect damage to critical biological components, including DNA, proteins, and cellular membranes. The harmful effects of radiation can lead to both somatic and hereditary damage, with long-term consequences, such as cancer development. The development and use of radioprotectors are crucial in mitigating these effects, providing a means to protect healthy tissues from radiation, while allowing therapeutic treatments like radiotherapy to target malignancies effectively. Continued research into radioprotective agents and their mechanisms remains essential for minimizing the risks associated with radiation exposure and ensuring the safe use of ionizing radiation in various applications. The development of radioprotective agents faces several significant challenges and holds promising future perspectives. One of the primary challenges is achieving effective protection against radiation damage without introducing toxic side effects, especially at higher doses. Ensuring that these agents are both efficacious and safe is essential, particularly in clinical settings. Another difficulty lies in achieving selectivity; ideally, radioprotectors should shield only healthy tissues without diminishing the therapeutic impact of radiotherapy on cancer cells. Stability and bioavailability also pose challenges, as these agents need to be stable and readily administrable, with sufficient bioavailability to act swiftly and effectively in the body. Looking forward, there will be future research exploring several promising directions. Novel molecular approaches focus on enhancing the body’s natural antioxidant defenses and activating DNA repair pathways, offering new potential for effective radioprotectors. Additionally, natural radioprotectors and plant-derived compounds are gaining interest, as they may provide protection with fewer adverse effects. Advances in delivery technologies, such as nanomedicine and encapsulation techniques, could improve the targeting of radioprotectors to specific tissues, maximizing their protective effects. Another promising avenue is the development of radiomitigators—agents that can be administered after radiation exposure to promote repair and recovery of damage. These agents could be especially valuable for mitigating unexpected or accidental radiation exposures. Addressing these challenges and harnessing these emerging strategies is critical for advancing the field of radioprotection and enhancing the safety and effectiveness of radiation applications in medicine and industry.

## Figures and Tables

**Table 1:** Deterministic effects at the tissue level.

Tissue	Effect	Approximate Latency Period	Approximate Threshold (Gy)	Severe Effects Dose (Gy)	Cause
Hematopoietic system	Hemorrhagic infections	2 weeks	0.5	2.0	Leukopenia Thrombocytopenia
Immune system	Immunosuppression Systemic infection	A few hours	0.1	1.0	Lymphopenia
Gastrointestinal system	Dehydration Malnutrition	1 week	2.0	5.0	Injury to the intestinal epithelium
Skin	Desquamation	3 weeks	3.0	10.0	Damage to the basal layer
Testicles	Sterility	2 months	0.2	3.0	Cellular aspermatism
Ovary	Sterility	<1 month	0.5	3.0	Interphase death of the oocyte
Lungs	Pneumonia	3 months	8.0	10.0	Alveolar barrier failure
Lens	Cataracts	>1 year	0.2	5.0	Maturation failure
Thyroid	Metabolic deficiencies	<1 year	5.0	10.0	Hypothyroidism
Central nervous system	Central nervous system	Central nervous system	15.0	30.0	Demyelination and vascular damage

**Table 2:** Decalogue for the ideal radioprotector [99].

**Characteristics**
Provides significant protection against radiation effects. Broadly protects healthy tissues and organs. Has an easy, preferably non-invasive, administration route. Contains a stable active product and compound formulation. Is compatible with other drugs and nutrients. Has an acceptable toxicity profile. Exhibits no intrinsic toxicity. Offers long-lasting protection. Has no positive effects on tumors in cancer therapy. Is cost-effectiveness for clinical use.

## Abbreviations

DNA	deoxyribonucleic acid
DSB	double-strand break
LET	Linear Energy Transfer
MeSHs	Medical Subject Headings
miRNA	microRNA
NO	nitric oxide
NOS	nitric oxide synthase
NTE	non-targeted effects
ONOO¯	peroxynitrite anion
RNA	ribonucleic acid
RNS	reactive nitrogen species
ROS	reactive oxygen species
RSH	sulfhydryl compounds
SSB	single-strand break

## References

[1] Jensen F. (1995). 100 years of X-rays. Med. Mundi.

[2] Furdui C.M. (2014). Ionizing radiation: Mechanisms and therapeutics. Antioxid. Redox Signal..

[3] Danyo E.K., Ivantsova M.N., Selezneva I.S. (2024). Ionizing radiation effects on microorganisms and its applications in the food industry. Food Raw Mater..

[4] Wikman-Svahn P. (2012). Radiation protection issues related to the use of nuclear power. Wiley Interdiscip. Rev. Energy Environ..

[5] Talapko J., Talapko D., Katalinić D., Kotris I., Erić I., Belić D., Škrlec I. (2024). Health Effects of Ionizing Radiation on the Human Body. Medicina.

[6] Averbeck D., Candéias S., Chandna S., Foray N., Friedl A.A., Haghdoost S., Sabatier L. (2020). Establishing mechanisms affecting the individual response to ionizing radiation. Int. J. Radiat. Biol..

[7] Pouget J.P. (2022). Basics of radiobiology. Nucl. Med. Mol. Imaging.

[8] Brieger K., Schiavone S., Miller F.J., Krause K.H. (2012). Reactive oxygen species: From health to disease. Swiss Med. Wkly..

[9] Obrador E., Salvador R., Villaescusa J.I., Soriano J.M., Estrela J.M., Montoro A. (2020). Radioprotection and radio-mitigation: From the bench to clinical practice. Biomedicines.

[10] Ibáñez B., Melero A., Montoro A., Merino-Torres J.F., Soriano J.M., San Onofre N. (2023). A Narrative Review of the Herbal Preparation of Ayurvedic, Traditional Chinese, and Kampō Medicines Applied as Radioprotectors. Antioxidants.

[11] Ward J.F. (1994). The complexity of DNA damage: Relevance to biological consequences. Int. J. Radiat. Biol..

[12] Alizadeh E., Orlando T.M., Sanche L. (2015). Biomolecular damage induced by ionizing radiation: The direct and indirect effects of low-energy electrons on DNA. Annu. Rev. Phys. Chem..

[13] Boudaïffa B., Cloutier P., Hunting D., Huels M.A., Sanche L. (2000). Resonant formation of DNA strand breaks by low-energy (3 to 20 eV) electrons. Science.

[14] Sanche L. (2005). Low energy electron-driven damage in biomolecules. Eur. Phys. J. D.

[15] Murray D., McEwan A.J. (2007). Radiobiology of systemic radiation therapy. Cancer Biother. Radiopharm..

[16] Von Sonntag C. (1987). The Chemical Basis of Radiation Biology.

[17] Nikjoo H., O’Neill P., Terrissol M., Goodhead D.T. (1999). Quantitative modelling of DNA damage using Monte Carlo track structure method. Radiat. Environ. Biophys..

[18] Von Sonntag C. (2006). Free-Radical-Induced DNA Damage and Its Repair.

[19] Mohamad O., Sishc B.J., Saha J., Pompos A., Rahimi A., Story M.D., Davis A.J., Kim D.W.N. (2017). Carbon ion radiotherapy: A review of clinical experiences and preclinical research, with an emphasis on DNA damage/repair. Cancers.

[20] Pfeiffer P., Goedecke W., Obe G. (2000). Mechanisms of DNA double-strand break repair and their potential to induce chromosomal aberrations. Mutagenesis.

[21] Wardman P. (2007). Chemical radiosensitizers for use in radiotherapy. Clin. Oncol..

[22] Hayes J.D., Dinkova-Kostova A.T. (2014). The Nrf2 regulatory network provides an interface between redox and intermediary metabolism. Trends Biochem. Sci..

[23] Riley P.A. (1994). Free radicals in biology: Oxidative stress and the effects of ionizing radiation. Int. J. Radiat. Biol..

[24] Hall E.J., Giaccia A.J. (2020). Radiobiology for the Radiologist.

[25] LaVerne J.A. (2000). Track effects of heavy ions in liquid water. Radiat. Res..

[26] Spinks J.W.T., Woods R.J. (1990). An Introduction to Radiation Chemistry.

[27] Mozumder A. (1999). Fundamentals of Radiation Chemistry.

[28] Jackson S.P., Bartek J. (2009). The DNA-damage response in human biology and disease. Nature.

[29] Huels M.A., Boudaïffa B., Cloutier P., Hunting D., Sanche L. (2003). Single, double, and multiple double strand breaks induced in DNA by 3−100 eV electrons. J. Am. Chem. Soc..

[30] Friedberg E.C., Walker G.C., Siede W., Wood R.D., Schultz R.A., Ellenberger T. (2006). DNA Repair and Mutagenesis.

[31] Nikjoo H., O’Neill P., Wilson W.E., Goodhead D.T. (2001). Computational Approaches for Determining the Spectrum of DNA Damage Induced by Ionizing Radiation. Radiat. Res..

[32] Lomax M.E., Folkes L.K., O’Neill P. (2013). Biological consequences of radiation-induced DNA damage: Relevance to radiotherapy. Clin. Oncol..

[33] Goodhead D.T., Thacker J., Cox R. (1993). Effects of Radiations of Different Qualities on Cells: Molecular Mechanisms of Damage and Repair. Int. J. Radiat. Biol..

[34] Alizadeh E., Sanche L. (2012). Precursors of solvated electrons in radiobiological physics and chemistry. Chem. Rev..

[35] Spotheim-Maurizot M., Mostafavi M., Belloni J., Douki T., Markovitsi D. (2008). Radiation Chemistry: From Basics to Applications in Material and Life Sciences.

[36] Le Caër S. (2020). Water Radiolysis: Influence of Oxide Surfaces on H_2_ Production under Ionizing Radiation. Water.

[37] Lushchak V.I. (2011). Environmentally induced oxidative stress in aquatic animals. Aquat. Toxicol..

[38] Reczek C.R., Chandel N.S. (2017). The two faces of reactive oxygen species in cancer. Nat. Rev. Cancer.

[39] Kirkland D., Marzin D. (2003). An assessment of the genotoxicity of 2-hydroxybenzylamine, a potential new cancer therapeutic agent, and hydrogen peroxide. Mutat. Res..

[40] Edge R., Truscott T.G. (2021). The reactive oxygen species singlet oxygen, hydroxy radicals, and the superoxide radical anion—Examples of their roles in biology and medicine. Oxygen.

[41] Buxton G.V., Greenstock C.L., Helman W.P., Ross A.B. (1988). Critical review of rate constants for reactions of hydrated electrons, hydrogen atoms and hydroxyl radicals (⋅OH/⋅O^−^) in aqueous solution. J. Phys. Chem. Ref. Data.

[42] Davies M.J. (2011). Myeloperoxidase-derived oxidation: Mechanisms of biological damage and its prevention. J. Clin. Biochem. Nutr..

[43] Ayala A., Muñoz M.F., Argüelles S. (2014). Lipid peroxidation: Production, metabolism, and signaling mechanisms of malondialdehyde and 4-hydroxy-2-nonenal. Oxid. Med. Cell. Longev..

[44] von Sonntag C., Schuchmann H.P. (1991). The elucidation of peroxyl radical reactions in aqueous solution. Angew. Chem. Int. Ed..

[45] Yin H., Xu L., Porter N.A. (2011). Free radical lipid peroxidation: Mechanisms and analysis. Chem. Rev..

[46] Radi R. (2013). Peroxynitrite, a stealthy biological oxidant. J. Biol. Chem..

[47] Narayanan P.K., Goodwin E.H., Lehnert B.E. (1997). α particles initiate biological production of superoxide anions and hydrogen peroxide in human cells. Cancer Res..

[48] Azzam E.I., de Toledo S.M., Little J.B. (2003). Oxidative metabolism, gap junctions and the ionizing radiation-induced bystander effect. Oncogene.

[49] Prise K.M., O’Sullivan J.M. (2009). Radiation-induced bystander signalling in cancer therapy. Nat. Rev. Cancer.

[50] Maier P., Hartmann L., Wenz F., Herskind C. (2021). Cellular Pathways in Response to Ionizing Radiation and Their Targeting for Tumor Radiosensitization. Int. J. Mol. Sci..

[51] Kirsch D.G., Diehn M., Kesarwala A.H., Maity A., Morgan M.A., Schwarz J.K., Bernhard E.J. (2018). The future of radiobiology. JNCI J. Natl. Cancer Inst..

[52] Demaria S., Formenti S.C. (2020). Role of T Lymphocytes in Tumor Response to Radiotherapy. Radiat. Res..

[53] Tang F.R., Loke W.K. (2015). Molecular mechanisms of low dose ionizing radiation-induced hormesis, adaptive responses, radioresistance, bystander effects, and genomic instability. Int. J. Radiat. Biol..

[54] Morgan W.F., Sowa M.B. (2021). Non-Targeted Effects of Ionizing Radiation: Implications for Risk Assessment and the Protective Effects of Radioprotectors. Int. J. Radiat. Biol..

[55] Allen C., Her S., Jaffray D.A. (2017). Radiotherapy for cancer: Present and future. Adv. Drug Deliv. Rev..

[56] Stadtman E.R., Levine R.L. (2003). Free radical-mediated oxidation of free amino acids and amino acid residues in proteins. Amino Acids.

[57] van Gent D.C., Hoeijmakers J.H., Kanaar R. (2001). Chromosomal stability and the DNA double-stranded break connection. Nat. Rev. Genet..

[58] Mavragani I.V., Nikitaki Z., Georgakilas A.G. (2022). Ionizing radiation and complex DNA damage: From prediction to detection challenges and biological significance. Cancers.

[59] Laayoun A., Lhomme J., Berger M., Cadet J. (2021). Protection against radiation damage to DNA bases. Radioprotectors.

[60] Cadet J., Bellon S., Douki T., Frelon S., Gasparutto D., Muller E., Sauvaigo S. (2004). Radiation-induced DNA damage: Formation, measurement, and biochemical features. J. Environ. Pathol. Toxicol. Oncol..

[61] Ponomarev D.B., Stepanov A.V., Seleznyov A.B., Ivchenko E.V. (2023). Ionizing Radiation and Inflammatory Reactions: Formation Mechanisms and Implications. Biol. Bull..

[62] Chatzipapas K.P., Papadimitroulas P., Emfietzoglou D., Kalospyros S.A., Hada M., Georgakilas A.G., Kagadis G.C. (2020). Ionizing radiation and complex DNA damage: Quantifying the radiobiological damage using Monte Carlo simulations. Cancers.

[63] Fowler J.F. (2006). Development of radiobiology for oncology—A personal view. Phys. Med. Biol..

[64] Kamiya K., Ozasa K., Akiba S., Niwa O., Kodama K., Takamura N., Wakeford R. (2015). Long-term effects of radiation exposure on health. Lancet.

[65] Sankaranarayanan K. (2006). Estimation of the genetic risks of exposure to ionizing radiation in humans: Current status and emerging perspectives. J. Radiat. Res..

[66] Ainsbury E.A., Bouffler S.D., Dörr W., Graw J., Muirhead C.R., Edwards A.A., Cooper J. (2009). Radiation cataractogenesis: A review of recent studies. Radiat. Res..

[67] Chang D.S., Lasley F.D., Das I.J., Mendonca M.S., Dynlacht J.R. (2021). Stochastic, deterministic, and heritable effects (and some radiation protection basics). Basic Radiotherapy Physics and Biology.

[68] Sia J., Szmyd R., Hau E., Gee H.E. (2020). Molecular mechanisms of radiation-induced cancer cell death: A primer. Front. Cell Dev. Biol..

[69] Blakely E.A., Kleiman N.J., Neriishi K., Chodick G., Chylack L.T., Cucinotta F.A., Shore R.E. (2010). Radiation cataractogenesis: Epidemiology and biology. Radiat. Res..

[70] Johnke R.M., Sattler J.A., Allison R.R. (2014). Radioprotective agents for radiation therapy: Future trends. Future Oncol..

[71] Barendsen G.W., Van Bree C., Franken N.A. (2001). Importance of cell proliferative state and potentially lethal damage repair on radiation effectiveness: Implications for combined tumor treatments. Int. J. Oncol..

[72] Held K.D. (2006). Radiobiology for the Radiologist.

[73] Jiao Y., Cao F., Liu H. (2022). Radiation-induced cell death and its mechanisms. Health Phys..

[74] McMahon S.J., Prise K.M. (2019). Mechanistic modelling of radiation responses. Cancers.

[75] Mothersill C., Seymour C. (2022). Low dose radiation mechanisms: The certainty of uncertainty. Mutat. Res./Genet. Toxicol. Environ. Mutagen..

[76] Belli M., Tabocchini M.A. (2020). Ionizing radiation-induced epigenetic modifications and their relevance to radiation protection. Int. J. Mol. Sci..

[77] Muriel V., Serrano N. (2004). Mechanisms, models and risks of radiation carcinogenesis. Clin. Transl. Oncol..

[78] Shuryak I. (2019). Enhancing low-dose risk assessment using mechanistic mathematical models of radiation effects. J. Radiol. Prot..

[79] Jayashree B., Devasagayam T.P.A., Kesavan P.C. (2001). Low dose radiobiology: Mechanistic considerations. Curr. Sci..

[80] Averbeck D., Salomaa S., Bouffler S., Ottolenghi A., Smyth V., Sabatier L. (2018). Progress in low dose health risk research: Novel effects and new concepts in low dose radiobiology. Mutat. Res. Rev. Mutat. Res..

[81] Alamilla-Presuel J.C., Burgos-Molina A.M., González-Vidal A., Sendra-Portero F., Ruiz-Gómez M.J. (2022). Factors and molecular mechanisms of radiation resistance in cancer cells. Int. J. Radiat. Biol..

[82] Piotrowski I., Kulcenty K., Suchorska W.M., Skrobała A., Skórska M., Kruszyna-Mochalska M., Malicki J. (2017). Carcinogenesis induced by low-dose radiation. Radiol. Oncol..

[83] Barcellos-Hoff M.H., Lyden D., Wang T.C. (2013). The evolution of the cancer niche during multistage carcinogenesis. Nat. Rev. Cancer.

[84] Schöllnberger H., Stewart R.D., Mitchel R.E.J., Hofmann W. (2004). An examination of radiation hormesis mechanisms using a multistage carcinogenesis model. Nonlinearity Biol. Toxicol. Med..

[85] Dörr W. (2015). Radiobiology of tissue reactions. Ann. ICRP.

[86] Patt H.M., Tyree E.B., Straube R.L., Smith D.E. (1949). Cysteine protection against X irradiation. Science.

[87] Jagetia G.C. (2007). Radioprotective potential of plants and herbs against the effects of ionizing radiation. J. Clin. Biochem. Nutr..

[88] Block K.I. (2004). Antioxidants and cancer therapy: Furthering the debate. Integr. Cancer Ther..

[89] Allegra A.G., Mannino F., Innao V., Musolino C., Allegra A. (2020). Radioprotective agents and enhancers factors: Preventive and therapeutic strategies for oxidative induced radiotherapy damages in hematological malignancies. Antioxidants.

[90] Kalman N.S., Zhao S.S., Anscher M.S., Urdaneta A.I. (2017). Current status of targeted radioprotection and radiation injury mitigation and treatment agents: A critical review of the literature. Int. J. Radiat. Oncol. Biol. Phys..

[91] Shivappa P., Bernhardt G.V. (2022). Natural radioprotectors on current and future perspectives: A mini-review. J. Pharm. Bioallied Sci..

[92] Kamran M.Z., Ranjan A., Kaur N., Sur S., Tandon V. (2016). Radioprotective agents: Strategies and translational advances. Medicinal Res. Rev..

[93] Chaturvedi A., Jain V. (2019). Effect of ionizing radiation on human health. Int. J. Plant Environ..

[94] Wang K.X., Ye C., Yang X., Ma P., Yan C., Luo L. (2023). New insights into the understanding of mechanisms of radiation-induced heart disease. Curr. Treat. Options Oncol..

[95] Reisz J.A., Bansal N., Qian J., Zhao W., Furdui C.M. (2014). Effects of ionizing radiation on biological molecules—Mechanisms of damage and emerging methods of detection. Antioxid. Redox Signal..

[96] Bushberg J.T., Kroger L.A., Hartman M.B., Leidholdt E.M., Miller K.L., Derlet R., Wraa C. (2007). Nuclear/radiological terrorism: Emergency department management of radiation casualties. J. Emerg. Med..

[97] Obrador E., Salvador-Palmer R., Villaescusa J.I., Gallego E., Pellicer B., Estrela J.M., Montoro A. (2022). Nuclear and radiological emergencies: Biological effects, countermeasures and biodosimetry. Antioxidants.

[98] Smith T.A., Kirkpatrick D.R., Smith S., Smith T.K., Pearson T., Kailasam A., Agrawal D.K. (2017). Radioprotective agents to prevent cellular damage due to ionizing radiation. J. Transl. Med..

[99] Upadhyay S.N., Dwarakanath B.S., Ravindranath T., Mathew T.L. (2005). Chemical radioprotectors. Def. Sci. J..

[100] Gong L., Zhang Y., Liu C., Zhang M., Han S. (2021). Application of radiosensitizers in cancer radiotherapy. Int. J. Nanomed..

[101] Aliper A.M., Bozdaganyan M.E., Sarkisova V.A., Veviorsky A.P., Ozerov I.V., Orekhov P.S., Korzinkin M.B., Moskalev A., Zhavoronkov A., Osipov A.N. (2020). Radioprotectors. org: An open database of known and predicted radioprotectors. Aging.

[102] Nair C.K., Parida D.K., Nomura T. (2001). Radioprotectors in radiotherapy. J. Radiat. Res..

[103] Montoro A., Obrador E., Mistry D., Forte G.I., Bravatà V., Minafra L., Mishra K.P. (2023). Radioprotectors, Radiomitigators, and Radiosensitizers. Radiobiology Textbook.

[104] Siama Z., Zosang-Zuali M., Vanlalruati A., Jagetia G.C., Pau K.S., Kumar N.S. (2019). Chronic low dose exposure of hospital workers to ionizing radiation leads to increased micronuclei frequency and reduced antioxidants in their peripheral blood lymphocytes. Int. J. Radiat. Biol..

[105] Colevas A.D., Brown J.M., Hahn S., Mitchell J., Camphausen K., Coleman C.N. (2003). Development of investigational radiation modifiers. J. Natl. Cancer Inst..

[106] Rosen E.M., Day R., Singh V.K. (2015). New approaches to radiation protection. Front. Oncol..

[107] Mishra K.N., Moftah B.A., Alsbeih G.A. (2018). Appraisal of mechanisms of radioprotection and therapeutic approaches of radiation countermeasures. Biomed. Pharmacother..

[108] Moulder J.E., Cohen E.P. (2007). Future strategies for mitigation and treatment of chronic radiation-induced normal tissue injury. In Semin. Radiat. Oncol..

[109] Marrone A., Tran W.T. (2015). Cytotoxic agents and radiation therapy: Mechanisms of action and clinical applications. J. Radiother. Pract..

[110] Seed T.M. (2005). Radioprotectants: Current status and future prospects. J. Radiat. Res..

[111] Stasiłowicz-Krzemień A., Gościniak A., Formanowicz D., Cielecka-Piontek J. (2024). Natural guardians: Natural compounds as radioprotectors in cancer therapy. Int. J. Mol. Sci..

[112] Howard D., Sebastian S., Le Q.V.C., Thierry B., Kempson I. (2020). Chemical mechanisms of nanoparticle radiosensitization and radioprotection: A review of structure-function relationships influencing reactive oxygen species. Int. J. Mol. Sci..

[113] Gudkov S.V., Popova N.R., Bruskov V.I. (2015). Radioprotective substances: History, trends and prospects. Biophysics.

[114] Zhang Y., Huang Y., Li Z., Wu H., Zou B., Xu Y. (2023). Exploring natural products as radioprotective agents for cancer therapy: Mechanisms, challenges, and opportunities. Cancers.

[115] Esmaealzadeh N., Iranpanah A., Sarris J., Rahimi R. (2022). A literature review of the studies concerning selected plant-derived adaptogens and their general function in body with a focus on animal studies. Phytomedicine.

[116] Mira A., Gimenez E.M., Bolzan A.D., Bianchi M.S., López-Larraza D.M. (2013). Effect of thiol compounds on bleomycin-induced DNA and chromosome damage in human cells. Arch. Environ. Occup. Health.

